# Diagnostic Accuracy and Clinical Outcomes of ECG-Gated, Whole Chest CT in the Emergency Department

**DOI:** 10.1371/journal.pone.0061121

**Published:** 2013-04-16

**Authors:** Kelley R. Branch, Jared Strote, William P. Shuman, Lee M. Mitsumori, Janet M. Busey, Tessa Rue, James H. Caldwell

**Affiliations:** 1 Division of Cardiology, University of Washington, Seattle, Washington, United States of America; 2 Department of Emergency Medicine, University of Washington, Seattle, Washington, United States of America; 3 Department of Radiology, University of Washington, Seattle, Washington, United States of America; 4 Department of Biostatistics, University of Washington, Seattle, Washington, United States of America; S.G.Battista Hospital, Italy

## Abstract

**Purpose:**

The purpose of this study was to assess the diagnostic accuracy and one year prognosis of whole chest, “multiple rule out” CT for coronary artery disease (CAD) in Emergency Department patients.

**Methods and Findings:**

One hundred and two Emergency Department patients at low to intermediate risk of acute coronary syndrome (ACS), pulmonary embolism and/or acute aortic syndrome underwent a research 64 channel ECG-gated, whole chest CT and a standard of care evaluation. Patients were classified with obstructive CAD with either a coronary CT stenosis greater than 50% or a non-evaluable coronary segment. SOC and 3 month follow up data were used to determine an adjudicated clinical diagnosis. The diagnostic ability of obstructive CAD on CT to identify clinical diagnoses was determined. Patients were followed up for 1 year for cardiac events. Seven (7%) patients were diagnosed with ACS. CT sensitivity to detect obstructive CAD in ACS patients was 100% (95% CI 65%, 100%), negative predictive value 100% (96%, 100%), specificity 88% (80%, 94%), and positive predictive value 39% (17%, 64%). Pulmonary embolism and acute aortic syndrome were not identified in any patients. No cardiac events occurred in patients without obstructive CAD over 1 year.

**Conclusions:**

Whole chest CT has high sensitivity and negative predictive value for ACS with excellent one year prognosis in patients without obstructive CAD on CT. The frequency of pulmonary embolism or acute aortic syndrome and the higher radiation dose suggest whole chest CT should be limited to select patients.

ClinicalTrials.org #: NCT00855231

## Introduction

Patients who present to the Emergency Department (ED) with possible acute coronary syndrome (ACS) commonly have symptoms that suggest a larger differential diagnosis than ACS alone. The considerable overlap of symptoms for multiple potential life threatening disease, including pulmonary embolism (PE) and acute aortic syndrome (AAS), makes ED evaluation challenging and may result in missed diagnoses in up to 10% of these patients [1], [2], [3]. Whole chest computed tomography (CT) may be useful in these patients using a “Triple-” or “Multiple-Rule-Out” technique to evaluate coronary arteries, aorta, pulmonary vasculature, and intrathoracic structures in a single study. Evaluation of ACS by ECG-gated, multislice CT is highly accurate with a very high negative predictive value compared to the standard of care (SOC) [4], [5]. Further, ECG-gated, multislice CT has been shown to both reduce time in the ED and reduce patient costs [6], [7], [8]. While studies have primarily evaluated patients using dedicated cardiac CT, the total number of patients studied with whole chest CT reported is small [9], [10], [11] with few data on intermediate term prognosis [12], [13]. Further, use of whole chest CT in ED patients is controversial due to potential compromise of coronary artery image quality, the higher radiation dose, and the higher prevalence of incidental CT findings [14], [15]. Our study explored the diagnostic accuracy, radiation dose, extracardiac findings, and one year prognosis of whole chest CT in a cohort of low to intermediate risk ED patients.

## Methods

### Study Design and Patient Population

**Figure 1:**
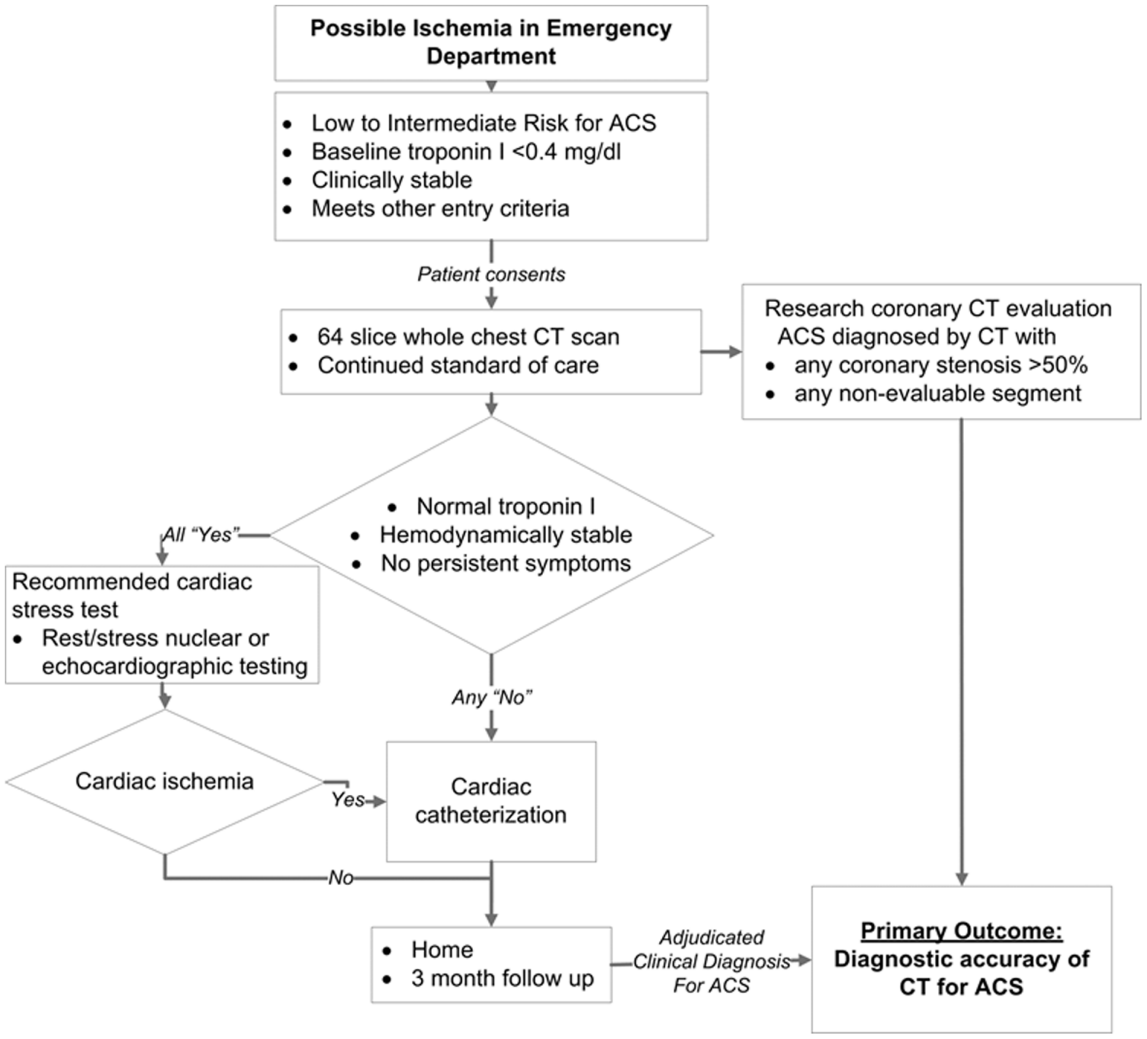
Clinical trial protocol and patient evaluation flowchart. ACS = acute coronary syndrome, CAD = coronary artery disease, CT = computed tomography.

This study used a prospective cohort study design. All sequential patients presenting to an urban, academic ED from July 2006 to May 2009 between the hours of 7∶00 AM and 5∶00 PM, Monday through Friday were screened for inclusion. The study protocol is attached as supporting information (Text S1). Inclusion criteria included age (male over 30 years or female over 40 years), presenting symptoms within 24 hours of ED arrival, and a low-to-intermediate clinical risk of ACS as determined by the clinical judgment of the treating ED physician. Exclusion criteria were ST segment elevation or dynamic ST changes, new left bundle branch block, troponin I greater than 0.4 mg/dl on presentation, estimated glomerular filtration rate of less than 40 ml/min/1.73 m^2^, pregnancy, clinical instability, ongoing bronchospasm, significant allergy to iodinated contrast, atrial fibrillation or markedly irregular heart rate, known coronary stenosis greater than 50%, or history of coronary revascularization. All patients that met eligibility criteria were approached by a trained research coordinator for informed consent (Figure 1). All patients underwent WRITTEN informed consent as a requirement of entry into the study. This study was approved by the University of Washington (UW) Human Subjects Division as a prospective trial (this is the UW institutional equivalent of the Institutional Review/Ethics Board). All patients were consented at the University of Washington.

#### CT technique

Whole chest CT scanning was performed in all patients using 64 channel scanner (LightSpeed VCT XT, GE Healthcare). A non-contrast calcium score was first obtained in all patients. Two CT techniques were used for patient scanning. From October 2006 to April 2007, only retrospective ECG-gated (R-CT) CTs were performed. Prospective ECG-triggered (P-CT) CTs were preferentially performed when they became available after that time. To prepare for CT scanning, metoprolol 50–100 mg orally or 5–15 mg intravenously was administered if baseline heart rate was greater than 60 bpm. Nitroglycerin 0.4 mg sublingual was given 3 minutes prior to CT scanning. Iodixanol 350 mg/dl contrast was injected with a dual power injector at 5 ml/sec using a triple phase contrast bolus of 1) 70 ml of contrast, 2) 50 ml of blended 70% contrast and 30% saline, and 3) 50 ml of saline. Scanning was performed during held inspiration in the cranial-caudal direction to cover from the lung apices to just below the diaphragm during a breath hold. CT source images were sent to a GE AW 4.3 workstation (GE Healthcare, Chalfont St. Giles, UK) for image processing and interpretation. Effective patient radiation dose was calculated using the dose-length product (DLP) generated from the CT scanner console and converted to millisievert (mSv) effective radiation dose by the adult conversion coefficient of the chest of mSv = DLP * 0.014 mSv/mGy/cm [16].

#### CT image review

Thoracic CT images were reviewed by board certified CT radiologists (>7 years experience) for thoracic findings. PE and AAS, which includes aortic dissection, were diagnosed by established criteria [17], [18], [19]. Cardiac images were reviewed independently by a cardiac CT Level III trained cardiologist and radiologist (>6 years cardiac CT experience each) who were blinded to the patient history and diagnosis. Any differences in image interpretation between reviewers were resolved by consensus. Image quality was scored on a four point scale from 1 (excellent) to 4 (non-evaluable) [20] using a modified 19 segment AHA model. Luminal coronary artery disease (CAD) stenosis was estimated qualitatively on an ordinal scale (No stenosis, 1–30%, 31–50%, 51–70%, 71–99%, 100%) and validated by quantitative coronary angiography analysis in vessels greater than 1 mm. To maximize CT sensitivity and minimize false negative CT studies, obstructive CAD on the CT scan was defined by at least one coronary segmental CT stenosis greater than 50% or a non-evaluable coronary segment. To ensure CT stenosis severity was not underestimated, non-evaluable segments were categorized as having greater than 70% stenosis for all analyses.

#### Clinical evaluation and adjudicated patient diagnosis

Pre-test probability of disease was assessed in two different ways. First, the subjective pre-test probability of ACS, PE, and AAS were determined on an ordinal scale (none, low, moderate, high probability) by the treating ED physician. Second, the TIMI Risk Score was used to estimate risk of major cardiovascular events. From October 2006 to April 2007, providers were given all CT findings except the results of coronary angiography for 22 patients. For the subsequent 80 patients, ED providers were told if coronary CT angiography was either 1) “positive” if there was one or more ≥30% coronary stenosis or 2) “negative” with all coronary stenoses <30%. No further coronary data were supplied. Irrespective of CT coronary angiography findings, all patients underwent a SOC evaluation for ACS, which recommended stress testing with imaging and/or cardiac catheterization testing based on current guidelines [21]. Further treatments were at the discretion of treating physicians. Nuclear stress testing and SPECT images were evaluated by standard criteria for ischemia [22], [23]. Invasive cardiac catheterization was performed in all patients with stress myocardial imaging with a summed difference score greater than 3 or a troponin I level >0.4 mg/dL during ED monitoring. The CT was not used as a basis for cardiac catheterization.

Patients were followed up by telephone contact at 1, 3, 6 and 12 months for any subsequent events, including repeat thoracic evaluation or hospitalizations. Outside records were obtained for additional patient evaluations, when possible. To arrive at an adjudicated ACS diagnosis, two physicians (K.B., J.C.), who were blinded to the coronary CT angiographic data, independently reviewed all available clinical testing and evaluations and 3 month clinical follow up. Each reviewer generated a prioritized adjudicated diagnosis list and the highest priority consensus diagnosis was considered the cause of the patient’s symptoms. ACS was diagnosed if 1) plasma troponin I was >0.4 mg/dL during hospitalization, 2) a nuclear stress test with a summed difference score was >3, 3) an echocardiographic stress test had new or worsening dyssynergy in at least 1 ventricular segment, or 4) unstable angina with a coronary stenosis >70% on invasive catheterization requiring revascularization.

#### Statistical analysis

Using the adjudicated diagnosis as the correct diagnosis, we determined the diagnostic ability of whole chest CT to identify ACS and other thoracic diseases. Empiric 95% confidence intervals were calculated for each point estimate (STATA software, College Station, TX). Receiver operator classification (ROC) curves and area under the curve (AUC) were calculated for >50% and >70% CT coronary stenosis thresholds and for the ordinal stenosis scale. Interobserver variability of CT coronary stenosis was calculated using the kappa statistic. Classification accuracy between P-CT and R-CT techniques was compared by relative probability estimates. A p-value of less than 0.05 was considered significant for all analyses. A STARD checklist for diagnostic accuracy studies is attached as a supporting information (Table S1).

## Results

**Figure 2:**
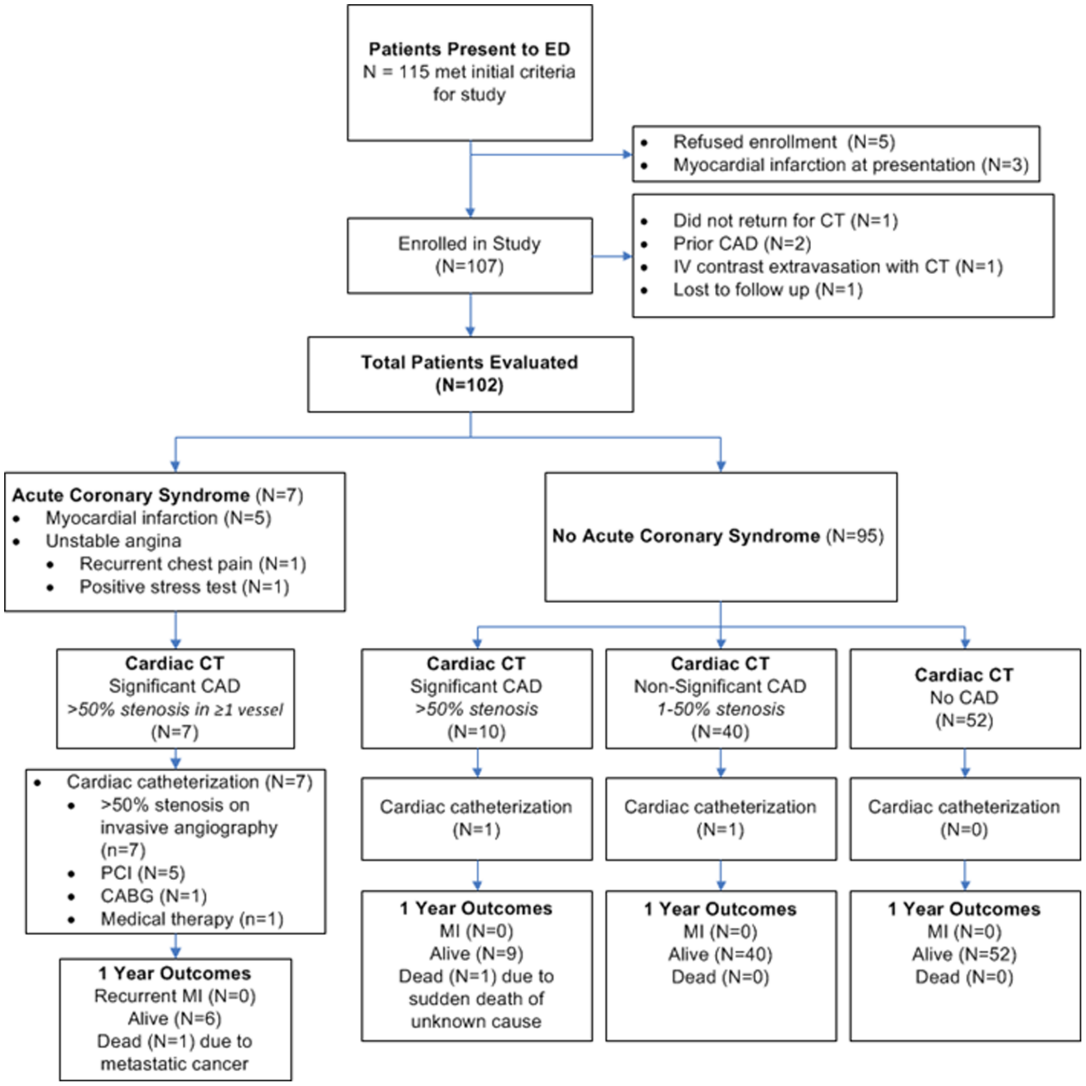
Patient enrollment and 3 month outcomes. CABG = coronary artery bypass grafting, CAD = coronary artery disease, IV = intravenous, MI = myocardial infarction, PCI = percutaneous coronary intervention.

From October 2006 to March 2009, 102 patients were enrolled (Figure 1). Baseline characteristics for the 102 analyzed patients are presented in Table 1. ED physician pre-test probability for thoracic disease categories are also shown in Table 1. Table 2 lists the primary symptoms that brought the patient to the ED as well as the SOC testing that was done for CAD. Of the 102 patients enrolled, 7 (7%) patients had ACS by adjudication (Figure 2). Five patients had myocardial infarction by elevated troponin I during SOC hospitalization and two patients were diagnosed with unstable angina. Two patients that were sent for invasive coronary angiography based on SOC clinical evaluation showed no obstructive CAD on CT or invasive angiography.

**Table 1:** Patient Baseline Characteristics.

Characteristic	N	Mean (95% CI) or %
Age (years)	102	54 (51, 57)
Male (%)	60	59%
Caucasian (%)	78	76%
Weight (kg)		86.5 (83, 90)
BMI		28.0 (27, 29)
TIMI ACS risk score		0.9 (0.7, 1.1)
Hypertension	43	42%
Dyslipidemia	39	38%
Diabetes	9	9%
Family history of premature CAD	39	38%
Recent tobacco	18	18%
Obesity	40	39%
Sedentary lifestyle	45	44%

ACS = acute coronary syndrome, CAD = coronary artery disease, BMI = body mass index.

**Table 2:** Presenting Symptoms and SOC Evaluation.

Presenting Symptoms*	N (%)
Chest Pain	97 (95%)
Syncope	5 (5%)
Palpitations	3 (3%)
Shortness of Breath	7 (7%)
Lightheadedness	1 (1%)
Back pain	2 (2%)
**SOC Testing for ACS**	**N (%)**
Stress Myocardial Perfusion SPECT	62 (61%)
- Treadmill	60 (59%)
- Adenosine	2 (2%)
Treadmill Echocardiogram	10 (10%)
Treadmill ECG Stress	1 (1%)
Invasive Coronary Angiography	9 (9%)
No Stress or Imaging Testing	23 (23%)
*Reasons:*	
- Physician preference	11 (11%)
- Lack of insurance	3 (3%)
- Did not return for stress testing	3 (3%)
- Left against medical advice	2 (2%)

* Patients may have more than one symptom at presentation. ACS = acute coronary syndrome, CAD = coronary artery disease, SOC = standard of care, SPECT = single positron emission computed tomography.

Overall CT coronary artery image quality was qualitatively excellent in 63% of patients. Agreement between readers for ordinal stenosis category on CT was good (kappa = 0.74). Twelve (12%) patients had at least one non-evaluable coronary segment. Two (2%) of the CT exams were entirely non-evaluable due to poor coronary contrast enhancement and image noise due to body habitus, respectively. Fourteen non-evaluable CT segments were present in all patients. Six (6%) patients had one non-evaluable segment and 4 (4%) patients had more than one. All of these patients were assumed to have a >70% stenosis. Reasons for the 14 non-evaluable CT segments were irregular heart beat (n = 5), coronary calcification (n = 4), poor contrast enhancement of distal vessels (n = 3), image noise due to large body habitus (n = 1), and patient motion during CT scanning (n = 1).

**Figure 3:**
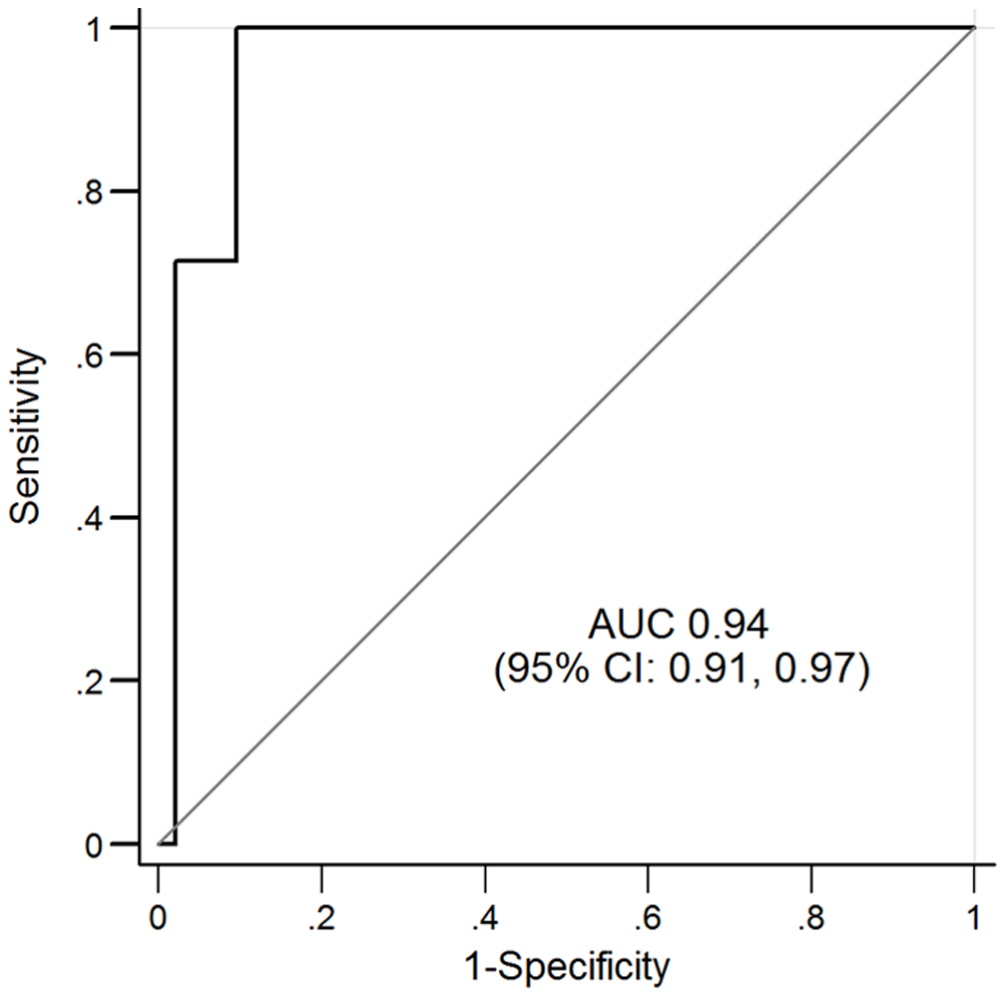
Empiric ROC curve for ordinal coronary CT stenosis quintiles compared to the adjudicated diagnosis of ACS. AUC = area under the curve.

**Figure 4:**
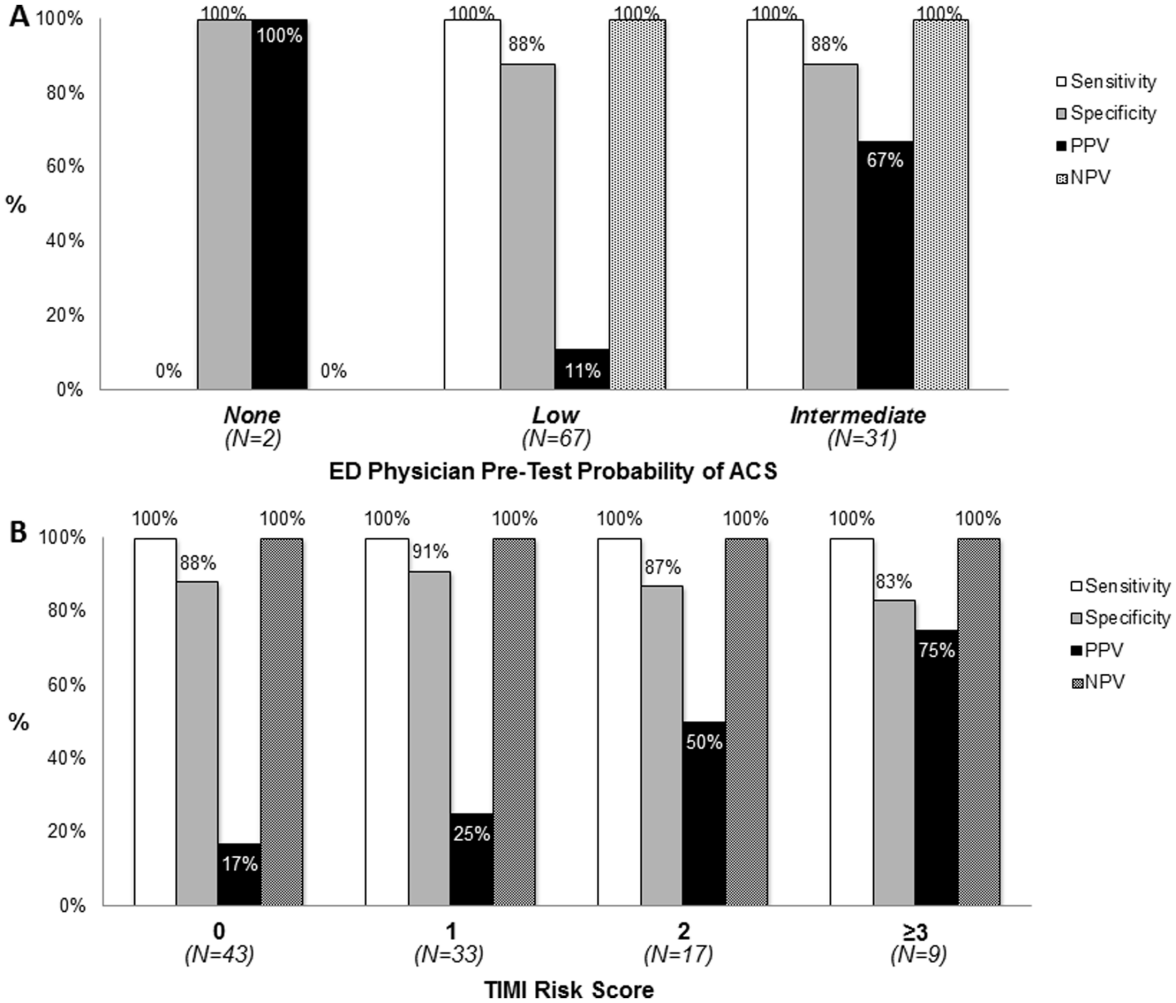
Patient-level diagnostic measures of CT by clinical pre-test probability of ACS (A) and by TIMI Risk Score (B). The pre-test probability of ACS (A) was determined by the clinical judgment of the ED physician caring for the patient. NPV = negative predictive value; PPV = positive predictive value.

The patient-level diagnostic ability of CT to diagnosis ACS is presented in Table 3. All 7 (7%) ACS patients had obstructive CAD on CT as well as >50% stenoses confirmed with invasive coronary angiography. Using the ordinal scale of CT stenosis, the ROC curve for CT had an AUC of 0.97 (95% CI: 0.92, 0.99; Figure 3) for diagnosing ACS. This was similar to the AUC using a binary 50% CT stenosis threshold (AUC 0.94, 95% CI 0.91, 0.94). Not surprisingly, using a 70% CT stenosis threshold showed a decreased sensitivity but an increased specificity (Table 3). In addition, the positive predictive value increased in relation to increasing clinical pre-test probability of ACS or TIMI Risk Score (Figure 4).

**Table 3:** Patient-Level Diagnostic Measures of CT for Acute Coronary Syndrome.

Obstructive CT Threshold	Sensitivity*Mean [95% CI]*	Specificity*Mean [95% CI]*	PPV*Mean [95% CI]*	NPV*Mean [95% CI]*
*50% Stenosis*	100% [59%,100%]	88% [80%, 94%]	39% [17%,64%]	100% [96%,100%]
*70% Stenosis*	86% [42%,100%]	95% [88%,98%]	55% [23%,83%]	99% [94%,100%]

Two CT scans were non-evaluable and patients were considered to have >70% stenosis. 95% CI = empiric 95% confidence limits, PPV = Positive predictive value; NPV = Negative predictive value.

There were no pulmonary emboli or aortic dissections identified by CT. Qualtitative ED provider pre-test probabilities for PE were none (28%), low (63%) and intermediate (9%). Pre-test probability for AAS were none (33%), low (64%) and intermediate (3%). No patients were deemed high probability for PE or AAS. Incidental findings on CT are listed in Table 4. Patients with ascending aortic aneurysm were referred to a cardiologist for routine screening of their aortas. The patient with bronchiolitis obliterans was referred to pulmonary medicine for additional care. Hiatal hernia by CT was diagnosed in 17 (17%) of patients and had a sensitivity of 20% (8%, 40%) and a specificity of 81% (70%, 89%) for the clinical diagnosis of gastroesophageal reflux disease.

**Table 4:** Non-Cardiac Findings.

CT Findings	N (%)	Follow Up Testing
Hiatal Hernia	17 (17%)	0
Pulmonary Nodules	10 (10%)	2*
Atelectasis in Lung Base	3 (3%)	0
Hepatic Cyst	3 (3%)	1^†^
Degenerative Changes In Spine	2 (2%)	0
Pericardial Effusion	2 (2%)	0
Calcified Lymph Nodes	2 (2%)	0
Enlarged Lymph Nodes	2 (2%)	0
Dilated Ascending Aorta	3 (3%)	3
Azygous Lobe	1 (1%)	0
Liver Hemanginoma	1 (1%)	1^†^
Hepatic Lesion	1 (1%)	0
Bronchiolitis Obliterans	1 (1%)	1
Kidney Cyst	1 (1%)	0
Calcified Asbestos-Related Plural Disease	1 (1%)	0
Thyroid Nodule	1 (1%)	0

Follow up imaging with ultrasound^†^ and repeat CT* suggested benign findings.

Mean effective radiation dose for the total CT examination (including the calcium score and timing bolus scan) was 17.8 mSv (95% CI 15.6, 19.9 mSv) with the mean CT scan dose of 15.2 mSv (13.2, 17.2 mSv). In the 47 patients that had R-CT technique, mean total dose was 27.2 mSv (25.4, 29.1 mSv) with CT mean dose of 24.4 mSv (22.8, 26.0 mSv). Total radiation dose was reduced 65% with P-CT technique to a mean 9.7 mSv (9.2, 10.2 mSv; p<0.00005). P-CT mean dose for the scan alone was 7.3 mSv (6.9, 7.8 mSv). There were no differences in any diagnostic measures between R-CT and P-CT techniques (p>0.5 for all diagnostic measures in Table 3).

Of the 85 patients without obstructive CAD on CT, no patients had an ACS event or death during 1 year follow up (Figure 2). One diabetic patient that had non-evaluable right coronary segments due to poor contrast enhancement died suddenly at 2 months. No other coronary CT stenosis was observed. She was not adjudicated to have ACS based on her SOC evaluation. Of the 7 patients adjudicated to have ACS, 5 (5%) had coronary stent placement, 1 (1%) had coronary artery bypass surgery, and 1 (1%) had medical treatment without coronary revascularization.

## Discussion

Our study showed a 100% sensitivity and negative predictive value for whole chest CT to detect ACS in a low to intermediate risk Emergency Department population. Although no pulmonary embolism or acute aortic syndrome was identified by CT or the SOC, other non-life threatening thoracic diseases were identified by CT. In patients without obstructive CAD on CT, one year prognosis of whole chest CT was excellent.

Whole chest computed tomography (CT) as a “Triple-” or “Multiple-Rule-Out” technique can evaluate coronary arteries, aorta, pulmonary vasculature, and thoracic structures in a single study. While this is attractive as a comprehensive thoracic evaluation in the ED population, there have been concerns with use of whole chest CT scanning as compared to a dedicated cardiac CT. For instance, some authors have concerns that whole chest CT technique could compromise coronary evaluation [24]. To date, there are few data on coronary evaluation using a whole chest CT technique [9], [10], [11], [25]. In the only other whole chest CT study to report complete diagnostic data, Johnson, et al reported a sensitivity of 97%, a specificity of 77% and NPV of 91% for ACS in 55 ED patients. In the largest study to date, Takakuwa, et al reported a high negative predictive value of 99.4% [11] for ACS in 201 ED patients, although no other diagnostic data were reported. Our study showed similarly high sensitivity and NPV of 100%.

All of these data are comparable to those of dedicated cardiac CT [5], [12], [26], [27], [28]. In the largest diagnostic performance data of dedicated cardiac CT study published study to date, Hoffman, et al compared a blinded cardiac CT examination to the SOC in the same patients [5], similar to our study design. Of the 289 patients studied, ACS prevalence was 8% and CT NPV and sensitivity were 100%, which are also very similar to our data. Thus, our study adds to the cumulative data, suggesting that the diagnostic ability of coronary CT angiography to “rule out” ACS with a high NPV does not appear to be compromised with a whole chest technique.

In contrast, the positive predictive value for ACS in our study was low at 39% due to the false positive CT rate from non-evaluable coronary segments as well as the lower risk patient population studied. Non-evaluable segments were present in 12% of patients studied and negatively affected the specificity and positive predictive value. Most other studies have shown lower rates of non-evaluable segments [29], [30]. The majority of non-evaluable segments (40%) were a result of irregular heart rates which may result from breath hold and whole chest CT techniques alone, though this is unlikely. Patient respiratory motion during scanning was rare (1%) but may have been due to the longer breath hold. Otherwise, coronary calcification and poor opacification of the distal vessels accounted for remainder the non-evaluable segments. In contrast, 63% of CT studies were deemed “excellent” or a “1” on a 5 point ordinal scale, which is similar data to our prior unrelated study [20]. Other studies with first and second generation 64 slice scanners have shown a similar range of “excellent” results [31], although other reports are much higher [32], [33]. Given the range of values, this discrepancy may lie in our more conservative, subjective interpretation of an “excellent” scan rather than decreased image quality of whole chest scanning. However, substandard and non-evaluable segments may result in hospital admission and potentially unwarranted invasive cardiac catheterization which would both increase costs and patient risk. Further studies investigating whether the false positive rate is indeed different with whole chest CT techniques appear warranted.

The overall low positive predictive value was also related to pre-CT patient risk. Most patients in our study were deemed “low risk”, which affects specificity and positive predictive values, although many were at “intermediate risk” (Figure 4). Consistent with Bayesian principles, positive predictive value of CT was related to higher clinical pre-test probability for ACS or with higher risk of cardiovascular events by the TIMI Risk Score (Figure 4). It is not surprising that higher risk patients with obstructive CAD on CT are more likely to have ACS and this is further evidence that optimal use of CT is in intermediate risk patients. Since CT “rules out” CAD in the majority of patients, patient selection may gravitate to the low risk population. Lower specificity and positive predictive value are challenging for whole chest and dedicated coronary CT and further clinical and technical developments are needed. Protocols that incorporate pre-test probabilities into determining suitability of patients for CT as well as which patients should proceed to further cardiac testing may be one options, but not currently available. We recently reported first-pass CT perfusion imaging techniques that when applied to the existing CT images may improve the ACS specificity to as high as 100% and the positive predictive value to 64% [34]. Developments like these are needed to further reduce the costs and risks of inappropriate hospital admissions of non-ACS patients for further testing of “obstructive” CAD.

Additional concerns for using a whole chest CT in ED patients include the higher radiation dose that is due to the longer thoracic coverage [14], [15] compared to dedicated cardiac CT. Radiation dose increases an average of 6% for each 1 cm increase in thoracic coverage [35]. The mean CT radiation dose in our study was 17.8 mSv using a first generation 64 slice scanner, with the highest dose in the 47 patients using a retrospective ECG-gated CT technique (mean 27.2 mSv). The use of prospective ECG-triggered CT reduced radiation dose 65% to a mean radiation dose of 9.7 mSv. While there are continuing improvements in CT design and technique to reduce radiation dose further [36], [37], [38], the small risk of radiation is concerning, especially in younger and female patients at higher potential risk for radiation-induced cancers [39]. In an observational study, whole chest CT did not change diagnostic yield compared to dedicated cardiac CT [25]. Further studies into appropriateness for different patient populations are needed.

Another potential concern about the use of whole chest CT involves incidental findings and false positive findings with the potential for significant unnecessary medical and patient costs. Non-cardiac findings were common, but only 8 (8%) of studies required follow up evaluation. Of these 4 (4%) were due to false positive findings (Table 4). However, a similar percentage of patients had other non-cardiac CT findings that either put the patient at higher risk for adverse outcomes or explained patient symptoms or that would have been missed otherwise. In our study, 4 patients had clinically important findings of thoracic aortic aneurysm (n = 3) and bronchiolitis obliterans (n = 1) that changed medical therapy. Further, hiatal hernia was common, occurring in 17% of patients, and highly specific (81%) for those patients that were eventually diagnosed with gastroesophageal reflux disease as the cause for their presenting ED symptoms. While hiatal hernia is not life threatening, identification by CT may lead to earlier appropriate treatment to decrease patient recidivism from recurrent chest pain.

The most important unanswered question is the proper patient selection for whole chest CT. At present, there are no current guidelines for selection of whole chest versus dedicated cardiac CT. Based on current data, we believe that clinical pre-test probability is the most important factor for whole chest CT selection. One important consideration is whether the patient will undergo CT scanning regardless. In our study, up to 62% of our patients would have had a non-gated CT angiogram as part of the SOC due to the ED provider’s qualitative pre-test risk of PE or AAS. The apparent maintained accuracy of coronary angiography makes whole chest ECG-gated CT an attractive option for these patients. However, PE and AAS were not seen in our study despite other authors having reported PE in 1–18% and aortic dissection in 0–2% of patients [11], [25], [40]. The major challenge is to define pre-test clinical suspicion cutoffs for non-ACS where the diagnostic benefits outweigh the potential costs of radiation and other concerns mentioned previously. While these issues will require further study, the current data reinforces that whole chest CT is a viable option for clinicians in the ED who want to rule out ACS while at the same time evaluate for other intrathoracic pathologies.

### Limitations

This study has several important limitations that require discussion. First, the number of patients is relatively small, but not unusual for a single center, pilot study. Second, not all patients received a cardiac stress test or invasive catheterization, so the adjudicated diagnosis is dependent on clinical evaluation and follow up for those patients. However, this reflects a real world evaluation where not all patients obtain stress testing after ED evaluation. Third, these studies were performed between 7AM and 5PM, Monday to Friday. This likely affects the cost savings of CT [8] but is unlikely to affect the diagnostic performance data presented. Finally, the study did not randomize patients to whole chest versus dedicated cardiac CT for comparison. However, the paired comparison decreased patient selection bias. A larger, multi-institutional study seems justified, especially to better establish CT diagnostic measures through a larger number of patients and to guide patient selection for a whole chest CT.

### Conclusions

Whole chest, ECG-gated CT angiography has a high sensitivity and negative predictive value for ACS for patients presenting to the ED. In addition, hiatal hernia on CT has a high specificity for the diagnosis of gastroesophageal reflux disease. Patients without obstructive CAD on CT have an excellent 1 year prognosis with no patients having major cardiovascular events. Given the higher radiation dose and questionable additive benefit of the longer CT coverage, whole chest CT should be only be used in selected patients. Further studies to identify the appropriate patient populations for whole chest CT appears warranted.

## Supporting Information

Table S1 **STARD checklist for reporting of studies of diagnostic accuracy.**
(DOC)

Text S1 **Study protocol.**
(DOCX)
