# Supplementary material for: Diagnostic Accuracy and Clinical Outcomes of ECG-Gated, Whole Chest CT in the Emergency Department
Source: PLoS One. 2013 Apr 16;8(4):e61121. doi: 10.1371/journal.pone.0061121 (PMC3629052; doi:10.1371/journal.pone.0061121)
Supplement: Text S1 — Study protocol. (DOCX) [file pone.0061121.s002.docx]

# Protocol outline

**Title of Study**: A Single Center Pilot Study to Determine Diagnostic Performance of 64 Slice CT Angiography in Emergency Department Patients with Chest Pain for Differentiation of Cardiac Causes from Aortic Dissection, Pulmonary Embolism, or other Non-cardiac Causes of Acute Chest Pain using GE LightSpeed VCT^TM^

**Acronym:** ACUTE CT Trial (Assessment of Acute Chest Pain Utilizing a Triple Rule Out Evaluation by CT).

**Rationale:**

Acute chest pain is a common and costly clinical condition accounting for over 5 million emergency department visits per year in the United States. In general, individuals presenting to the emergency department for chest pain require 6-24 hours of observation and multiple tests to differentiate pain from blocked heart arteries (i.e., acute or chronic coronary syndrome) from other non-cardiac sources.

Patient evaluation for cardiac causes typically includes sequential electrocardiograms and blood tests for heart enzymes to establish if a heart attack has occurred. Subsequently, cardiac stress testing or heart catheterization is required to firmly establish a diagnostic and treatment plan.

Non-cardiac but potentially life threatening illnesses can mimic cardiac symptoms and make an accurate diagnosis difficult, including a) blood clots in the lung vessels, or pulmonary embolism (PE), or b) a tearing of the vessel wall in the large artery supplying oxygenated blood to the body known as aortic dissection. These diagnoses are missed initially in the Emergency Room in up to 10% of patients presenting with acute chest pain. Failure to recognize these diseases often leads to delayed, unwarranted, or even detrimental medical treatments for these patients. Other non-cardiac causes of acute chest pain may include pneumonia, pleurisy, rib or other bone fracture, neuritis, or soft tissue injury.

Ideally, management of patients with acute chest pain would include a single test that is fast, easy to administer, widely and readily available at all times, and able to effectively differentiate many causes of acute chest pain. However, no single test currently exists.

CT is a widely available and standard imaging tool in Emergency Department (ED) that has been used for imaging applications throughout the body. CT angiography has been well studied and validated for identifying PE and aortic dissection when suspected, and is the preferred imaging modality for these diseases. Use of CT allows effective and timely evaluation for each of these potentially lethal diagnoses. CT can also effectively diagnose pneumonia, fracture, soft tissue injury, and may show signs suggesting pleurisy or neuritis. However, technical difficulties in imaging the moving heart and the size and motion of the small heart arteries (typically 4 to 1.5 mm and smaller) have been daunting for CT imaging.

Recent rapid advancement of multislice CT technology has allowed faster scanning times, better image resolution, and less intravenous contrast for visualization of structures. CT angiography holds the promise of enabling a non-invasive evaluation of the small coronary arteries that supply the heart. Four, 8, and 16 slice CT scanners have been used to imaging coronary arteries but poor spatial resolution and blooming artefact off calcified atherosclerosis has resulted in a significant number of coronary segments (up to 35% with 16 slice) that could not be evaluated using these scanners.

General Electric Healthcare has developed an innovative LightSpeed VCT. With the VCT scanner, 64-channels worth of data can be acquired in a single rotation, which allows cardiac CT data acquisition to be accomplished in the range of 5 seconds or 5 heart beats.

The newer, faster CT machine allows evaluation of coronary anatomy as well as atherosclerotic blockages with image resolution that approximates that of invasive coronary catheterization, the current gold standard test for coronary artery disease. **T**he 64 slice CT as an evaluation tool for patients presenting at ED with acute chest pain but with low to intermediate risk for angina would potentially allow rapid triage patients to 1) implement appropriate treatments, 2) ensure discontinuation of potentially harmful treatments, and 3) limit observation time and diagnostic testing in the acute setting.

In the ACUTE CT study, we propose using VCT as a first line imaging modality to evaluate patients presenting with acute chest pain in ED.

The hypothesis of the study is that

1. in low to intermediate CAD risk patients presenting with acute chest pain in ED, VCT examination will be able to exclude obstructive CAD as a cause for cardiac chest pain compared to routine evaluation and to accurately diagnose non-cardiac causes of acute chest pain by detecting presence of pulmonary embolus, aortic dissection, or other causes of chest pain.
2. the findings of VCT examination are able to accurately triage patients for proper management strategy
3. VCT examination will be able to reduce cost of and time required for managing patients with acute chest pain.

**Primary Objective:** To evaluate the diagnostic performance of VCT for differentiation of cardiac from non-cardiac causes of acute chest pain in ED to determine presence or absence of 1) significant blockages in the coronary arteries that supply the heart, 2) evidence of dissection of the aortic tissues, and 3) obstructive blood clots in the pulmonary arteries when compared to standard care information.

**Secondary Objectives:**

- To evaluate impact of the VCT findings on patient management.
- To evaluate the cost of VCT for determining the aetiology of acute chest pain when compared to the standard care.

**Number of Subjects Planned:** 150

**Inclusion Criteria:** Subjects may be included in the study if they meet all of the following criteria:

**Inclusion Criteria**

- Male: 30-75 years old or Female: 45 -75 years old
- Acute chest pain onset within 24 hours
- No obvious cause for chest pain
- Low to intermediate risk of myocardial ischemia by TIMI Unstable Angina Score (<5)
- One or more cardiac risk factors
  - Hypertension
  - Dyslipidemia
  - Diabetes
  - Family history – 1^st^ degree relative with CAD or cardiac event less than age 55 years
  - Current or recent smoker (<1 month)
  - Morbid obesity but BMI≤40
  - Sedentary lifestyle
- Able and willing to comply with study procedures and signed and dated informed consent
- For a female patient, she must be either surgically sterile (has had a documented bilateral oophorectomy and/or documented hysterectomy), or post-menopausal (cessation of menses for more than 1 year), or if of childbearing potential the results of a serum or urine human chorionic gonadotropin pregnancy test, performed within 36 hours of administration, must be negative.

**Exclusion Criteria:** Subjects must be excluded from participating in this study if they meet any of the following criteria:

1. Has known allergies to iodinated contrast agent, including but not limited to anaphylactoid or cardiovascular reactions, laryngeal edema, or bronchospasm
2. ST segment elevation or dynamic ST changes on ECG
3. Elevated cardiac enzymes (troponin I >2 mg/dl, CK-MB index > 9%)
4. Has atrial fibrillation/flutter or any irregular heart rhythm considered by the investigator to interfere with temporal acquisition of cardiac CT images.
5. Creatinine level >1.8 mg/dl
6. Pregnant or lactating female
7. Hemodynamic or respiratory instability as determined by the ED physician
8. Clinically active, serious, life-threatening disease with a life expectancy of less than 2 months.
9. Not willing/unable to discontinue metformin (eg, Glucophage^®^) at the time of the study procedure. Metformin must be withheld for at least 48 hours post-procedure and be restarted only after the subject’s renal function has been evaluated and it is deemed safe to resume metformin.
10. Previously included in this study.
11. Received a non-FDA approved drug within 30 days prior to the administration of the investigational product, or is scheduled to receive one during the administration of the investigational product, or within 72 hours afterward.
12. Moderate to severe reactive airway disease requiring >1 inhalor or prednisone or patient is actively wheezing on physical examination.

**Study Design:**

This study is designed as a single-centre trial to determine diagnostic value of VCT for differentiating cardiac from non-cardiac causes of acute chest pain in ED. Approximately 150 patients will be enrolled over a period of approximately 15 months.

The patient enrolment and study procedures are illustrated in the study flow charter, Figure 1.

Figure 1: Study Flow charter

Patients with acute chest pain presenting in ED

Initial Management by ED staff

- Clinical history
- ECG
- Chest X-ray
- IV line establishment and routine blood draw
- Medical therapy initiated

No or patient refuse

Standard care

No patient F/U

Eligible for enrollment?

Yes and patient consent

- Detailed history by research staff
- Additional blood for testing
- CT data available for clinical use when it is clinically needed
- The axial images and reconstructed image sets will be sent to core-lab for blinded evaluation

VCT scan

Two consensus readers review all clinical information and make diagnosis

- Routine care by primary physicians
- Additional imaging or lab tests
- Collection of medication, discharge diagnoses and cost information

Patient follow-up

**Study Imaging Tests:** Chest CT and cardiac CTA.

**CT Imaging Procedures:**

***Figure 2*** Outlines the CT scanning protocol.

*N*TG = nitroglycerin

*Subject preparation*

- Explain the test to the subject.
- Position the subject supine on the CT table with feet first.
- If the heart rate is >70 bpm, an IV bolus of metoprolol 2.5-5mg IV or oral dose of 100 mg PO metoprolol is given and repeated to achieve a heart rate <65 or until hypotension (systolic blood pressure <100 mmHg or the patient has lightheadedness or symptoms of hypoperfusion) limits further treatment
- A single dose of nitroglycerin of 0.4 mg given sublingually will be administered just prior to entering the CT gantry to dilate the coronary arteries for imaging if SBP > 100 mmHg
- Instruct and practice proper breath-holding with the subject using hyperventilation (take a breath in, blow the air out, take another breath in, blow the air out, and take a final breath in and hold the breath) and take note of the heart rate while they are holding their breath (oxygen can be given to the subjects who have difficulty holding their breath).

*ECG Leads and Power Injector Connection*

ECG leads should be placed in the appropriate location with firm connection. The subject’s arms must be raised above the head in order to appropriately position the leads: black – left chest below clavicle, white – right chest below clavicle, red – left abdomen below 6^th^ rib, and green – right abdomen below ribs (if it is available).

To confirm a good connection, check the ECG tracing on the monitor on the scan console.

Visipaque 320 mg-I/mL will be administered via power injector. Load power injector with 120 mL of the contrast agent. Approximately 70 mL saline must be loaded in the second barrel. The power injector must be securely connected to the subject’s IV line.

*Calcium Scan*

#### EKG-gated calcium scoring during single breath hold will be obtained using 2.5 mm slice thickness.

*Timing Test Bolus*

A test bolus is used to determine scan delay time.

Figure 1 A. Time Bolus Tracking Location; B. Left Main Artery Take Off


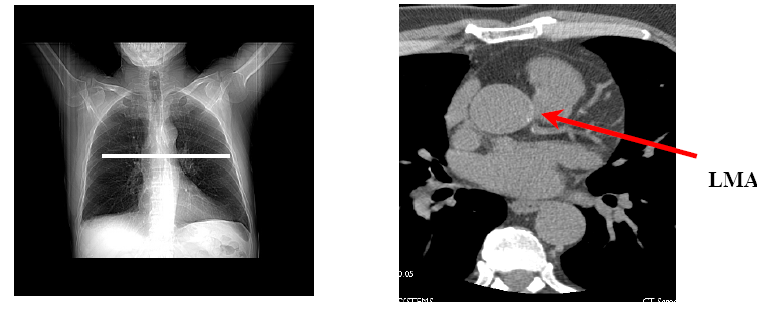


As illustrated in Figure3, to perform timing bolus, a tracking line is placed at the base of the heart (A), usually 1 cm below carina. On the axial view, this location corresponds to root of aorta near left main artery takeoff (B).

The timing test bolus protocol is described in Table 1.

| Table 1 Test Bolus Protocol | |
| --- | --- |
| Monitor Location | Aorta root near left main take-off (see picture below) |
| Scan type | Axial |
| Rotation time | 0.5 sec |
| Detector Collimator | 5 mm |
| Slice thickness | 5 mm |
| Interval | 0 |
| kV | 120 |
| MA | 80 |
| Pre-scan delay | 5 sec |
| Number of slices | 12-14 with 2 second cycle time |
| IV contrast volume and type | Visipaque 20 mL / 20 mL saline flush if dual barrel injector |
| Injection rate | 5mL/sec |

Once all of the test bolus images are reconstructed, the region of interest (ROI) will be selected and the measurement of contrast attenuation on each slice will be performed.

Figure 4 Example of a Graph Image

The measurement values are used to generate graph images. An example of a graph image is shown in Figure4. In this case, the peak of the curve is at the 7th tic mark on the line graph. Each tic mark represents 2 seconds with the peak of the curve occurring at 14 seconds. Since the initial pre-scan delay (or prep group delay) is 5 seconds, the time it takes for the contrast to reach the peak enhancement at the root of the aorta near left main artery takeoff is 14 + 5 = 19 seconds.

*Contrast administration*

Once appropriate scan parameters have been determined, the power injector will be programmed. The contrast agent will be administered using the injection protocol shown in Table 2

| Table 2 Visipaque Administration for Double-Barrel Power Injectors | | | |
| --- | --- | --- | --- |
| Phase | Product Name | Main Volume (mL) | Injection Rate (mL/Sec) |
| 1^st^ phase contrast | Visipaque 320 mg-I/mL | 70 | 5 |
| 2^nd^ phase mixture | 70% Visipaque and 30% 0.9% sodium chloride solution | 50 | 5 |
| 3^rd^ phase saline | 0.9% sodium chloride | 50 | 5 |

*Scan Parameters*

Scan parameters are set according to manufacturer recommendations/specifications. The typical range of the scan parameters is summarised in Table 3.

| Table 3 Scan Parameters for CT Scanner | |
| --- | --- |
|  | **64 – Slice Technology** |
| Application | Triple Rule out |
| Specific anatomic region | Chest |
| kV | 120 |
| MA | 400 – 800 (patient weight dependent) |
| Scan coverage | Top of lung to bottom of heart |
| Image order | Craniocaudal |
| Rotation time | 0.35 sec |
| Detector Collimator | 64 x 0.625 mm |
| Slice Thickness | 0.625 mm |
| Pitch | 0.2 mm – variable based on HR |
| Feed / Rotation (mm) | 8 –12 mm |
| Reconstruction interval | ≤0.5 mm |
| IV Contrast Volume and Type | 100 mL Visipaque / 70 cc saline flushing |
| Injection Rate | 5 cc/sec |

**CT Image Sequestering and Data Release**

The acquired CT images will be sent from the CT console to a secure server site after image acquisition. This site will not be accessible by anyone but the investigators and subjects will be de-identified for review. Within 48 hours, the CT images will be reviewed as outlined below. After review, if a non-cardiac life-threatening illness is identified on the CT scan, the physicians caring for the subject will be informed of the finding. Life threatening illnesses include 1) aortic dissection, 2) pulmonary embolism, 3) pneumothorax, or 4) esophageal rupture.

If the physicians caring for the patient believe that a CT scan is clinically indicated, the raw CT images will be released for routine evaluation by the on-call radiologist. Our findings will not be revealed to the radiologist or the physicians caring for the patient. However, any cardiac findings are NOT to be revealed to reduce bias of patient treatment based on the CT data.

**CT Image Evaluation/Procedures*:***

Two blinded readers who are qualified by their clinical training will be selected for evaluation of the CT images independently. All blinded reviewers must have no financial conflict. The independent image evaluation will be performed at a central imaging core-lab. Planar, cross sectional non-contrast and contrast CT images are compiled for analysis using GE AW 4.2 or higher workstation.

- Cross sectional, longitudinal, and curved planar CTA images will be reconstructed. Multiplanar images are created and reconstructed into a 3 dimensional rendering using the GE algorithms.
- Non-cardiac causes of acute chest pain will be evaluated and recorded, including pulmonary embolus, aortic dissection, lung parenchyma abnormalities, mediastinal abnormalities, other aortic abnormalities, rib fracture, pleurisy, etc.
- Coronary calcium scoring is calculated by the Agatston method, as well as volumetric and mass measurements.
- A 21 segmental ACC/AHA model of the coronary tree will be used for the coronary evaluation.
- For the coronary evaluation, two independent readers must review at least 10 imaging segment phases of images to determine:
- Overall image quality from single best phase for each of the 15 coronary segments in vessels 1.5 mm in diameter or larger.
- Coronary artery supply dominance and coronary anomalies
- Coronary artery segmental interpretability and vessel size down to 1.5 mm
- Presence or absence of coronary artery stenosis/occlusion
- Presence or absence of coronary plaque and characterizing vessel segment plaque as non-calcified, mixed, and calcified or combinations thereof. Non-calcified is defined as less than 40 HU, mixed is 40-150 HU, calcified is greater than 150 HU. A single lesion will be chosen for HU measured: that lesion showing greatest degree of negative or positive remodeling. Further, the segment extent of involvement will be visually graded as trace plaque (less than 10% of length of the vessel segment involved), mild plaque (10-25%), moderate (25-50%), or severe (50-100%).
- If a coronary stenosis >30% is suspected, CT quantitative coronary angiography analysis (QCA) is performed on the lesion to calculate minimal vessel lumen diameter and luminal area. Percent stenosis is calculated based on the proximal coronary considered non-significantly diseased divided into the minimal diameter and area of the lesion.

**Laboratory Analysis:**

- Patients undergo routine lab evaluation that includes complete blood count, comprehensive metabolic analysis (a.k.a., SMA-12), PT and PTT, troponin I/CPK/CK-MB/myoglobin every 8 hours, and lipid panel.
- Approximately 20 cc of blood will be collected into 2 red top tubes for additional testing. The blood will be analyzed for high sensitivity C-reactive protein, apolipoprotein A and B, d-dimer, hemoglobin A1C, and erythrocyte sedimentation rate.

**Treadmill Stress Testing Analysis:**

When clinically indicated, data from subjects undergoing treadmill stress testing will be assembled. Data points include treadmill duration, METS estimation, ECG ST segment measurements and morphology, and clinical symptoms. Data will be assembled and read independently by by two observers who will record disagreements but attempt to reach consensus. The stress test will be considered positive for ischemia if established criteria are met.

**Radionuclide Stress Testing:**

If a stress test with cardiac imaging is deemed necessary by the patient’s primary care team, a pharmacologic stress test will be administered. Either SPECT or PET will be used. Dipyramidole and adenosine are short acting arterial vasodilators that dilates coronary arteries to shunt blood flow away from a region supplied by a partially blocked coronary artery. To measure baseline myocardial blood flow, 20 mCi of ^99m^Tc- tetrofosmin or 20 mCi of ^13^N-ammonia will be injected IV as a bolus followed by dynamic image acquisition for a total 10 minute scan time. After approximately 30 minutes for radiotracer decay, stress testing with IV dipyramidole or adenosine begins. At approximately 6 minutes, a PET scan is acquired using 20 mCi of ^13^N-ammonia or ^99m^Tc with another 20 minutes of scan time. For SPECT, scanning occus within 20 min. Qualitative and quantitative myocardial blood flow as well as cardiac function will be tabulated for comparison. The images will be reviewed by two consensus readers.

**Coronary Angiography Image Analysis:**

If invasive coronary angiography is performed on a clinical basis, the digital coronary angiograms will be surveyed independently by two expert readers blinded to the patient history and coronary stenoses deemed >30% are evaluated by quantitative coronary angiography (QCA). At least 2 orthogonal diastolic views are used for each lesion. Automated vessel border detection is performed (ADAC/Philips, Bothell, WA) and luminal diameter both proximally and distally as well as within the lesion is measured; percent diameter stenosis is then calculated. The region of the myocardium supplied by the artery is estimated from the distribution of the vessel and its branches onto a polar map plot.

**Cost Analysis:**

After hospitalization, the total cost will be estimated by total charges as established through the hospital billing office and physicias billing office. These costs will be compiled and compared to the current charge for a thoracic CT scan. A fictive cost will then be determined base on diagnosis and treatment using the CT results and that will be compared to the actual cost incurred by the patient without the CT results . The risk/benefit ratio will be compiled using the risk of complications due to the CT scan (such as renal insufficiency) to the diagnostic benefits of the scan.

**Primary Efficacy Endpoint(s):**

Predictive values of VCT findings on differentiating cardiac from non-cardiac causes of acute chest pain in ED for determining 1) significant blockages in the coronary arteries that supply the heart, 2) evidence of dissection of the aortic tissues, 3) obstructive blood clots in the pulmonary arteries when compared to standard care information, and 4) other non-cardiac causes of chest pain.

**Secondary Efficacy Endpoint(s):**

- Accuracy of VCT findings for triage patients to 1) implement appropriate therapy, 2) discontinue unnecessary/potentially harmful treatments, 3) limit observation time and diagnostic testing in the ED setting for acute chest pain.
- Potential cost saving using VCT to determine the aetiology of acute chest pain when compared to the standard care by changing patient management strategy.

**Safety Variables:** The following safety data will be collected and evaluated through the CT procedure:

- Unexpected adverse events
- Serious adverse events

**Gold Standard/Standard of Truth/Truth Panel:** Routine clinical information, including but not limited to, medical history, laboratory tests, ECG tests, other image tests and non-cardiac CT findings. To arrive at an adjudicated diagnosis, two board certified cardiologists independently reviewed all available evidence including history and physical, chest radiograph, non-cardiac CT findings, ECG’s, stress tests, cardiac catheterization, clinical follow up, and pharmacologic trials. Patients were categorized as ACS in the adjudicated diagnosis if 1) plasma troponin I >0.4 mg/dL during the index hospitalization, 2) a nuclear stress test with a summed difference score > 3, 3) an echocardiographic stress test with new or worsening dyssynergy in at least 1 ventricular segment, or 4) coronary stenosis >70% on catheterization. Each reviewer generated a prioritized adjudicated diagnosis list for each patient after the 3 month follow up data were available. Discrepancies between the reviewer’s lists were resolved by consensus. The highest priority consensus diagnosis was considered the cause of the patient’s symptoms for data analysis.

**Statistical Methods:** The primary outcome is the diagnostic performance of CT compared to the standard of care to diagnose acute coronary syndrome (ACS). The classification accuracy of coronary CT to an adjudicated diagnosis for ACS. The diagnostic measures (e.g., sensitivity, specificity) for the CT diagnostic test for “significant CAD” (>50% stenosis) will be determined using the adjudicated diagnosis for ACS as the correct diagnosis. A >70% CTA stenosis will also be tested as a binary variable. Empiric confidence intervals will be calculated for each point estimate. Receiver operator classification (ROC) curves and area under the curve (AUC), both binary and empiric, will also be calculated. Classification accuracy between ECG-gating techniques (i.e., P-CT and R-CT) will be compared by relative probability estimates.
